# Atomic scale imaging of competing polar states in a Ruddlesden–Popper layered oxide

**DOI:** 10.1038/ncomms12572

**Published:** 2016-08-31

**Authors:** Greg Stone, Colin Ophus, Turan Birol, Jim Ciston, Che-Hui Lee, Ke Wang, Craig J. Fennie, Darrell G. Schlom, Nasim Alem, Venkatraman Gopalan

**Affiliations:** 1 Department of Materials Science and Engineering and Materials Research Institute, Pennsylvania State University, University Park, Pennsylvania 16802, USA; 2 National Center for Electron Microscopy, Molecular Foundry, Lawrence Berkeley National Laboratory, Berkeley, California 94720, USA; 3 Department of Chemical Engineering and Materials Science, University of Minnesota, Minneapolis 55455, USA; 4 Department of Materials Science and Engineering, Cornell University, Ithaca, New York 14853, USA; 5 Materials Characterization Laboratory, Materials Research Institute, Pennsylvania State University, University Park, Pennsylvania 16802, USA; 6 School of Applied and Engineering Physics, Cornell University, Ithaca, New York 14853, USA; 7 Kavli Institute at Cornell for Nanoscale Science, Ithaca, New York 14853, USA

## Abstract

Layered complex oxides offer an unusually rich materials platform for emergent phenomena through many built-in design knobs such as varied topologies, chemical ordering schemes and geometric tuning of the structure. A multitude of polar phases are predicted to compete in Ruddlesden–Popper (RP), *A*_*n*+1_*B*_*n*_O_3*n*+1_, thin films by tuning layer dimension (*n*) and strain; however, direct atomic-scale evidence for such competing states is currently absent. Using aberration-corrected scanning transmission electron microscopy with sub-Ångstrom resolution in Sr_*n*+1_Ti_*n*_O_3*n*+1_ thin films, we demonstrate the coexistence of antiferroelectric, ferroelectric and new ordered and low-symmetry phases. We also directly image the atomic rumpling of the rock salt layer, a critical feature in RP structures that is responsible for the competing phases; exceptional quantitative agreement between electron microscopy and density functional theory is demonstrated. The study shows that layered topologies can enable multifunctionality through highly competitive phases exhibiting diverse phenomena in a single structure.

## Abstract (web summary)

Competing phases in layered complex oxides are believed to be relevant for emergent phenomena, which still await to be witnessed. Here, Stone *et al*. report direct atomic-scale imaging of a multitude of polar phases in Ruddlesden-Popper oxide thin films, exhibiting diverse phenomena in a single structure.

## Graphical abstract

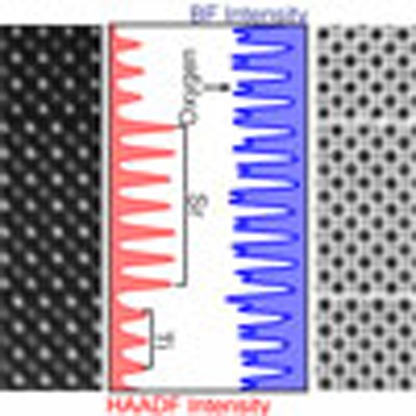

Complex oxides possess a rich array of properties^1^,^2^,^3^,^4^,^5^ that are closely linked to their atomic structure. Small changes in atom positions within a unit cell can lead to large changes in elastic, electronic and optical properties. While much of the focus thus far has been on simple perovskites, a survey of the inorganic crystal structure database (https://icsd.fiz-karlsruhe.de/) reveals that of the nearly 55,000 complex oxides listed today, >99% are layered structures; surprisingly, only ∼2% of these compounds have been explored. However, recent advances in unit-cell level control in the epitaxial growth of thin films and heterostructures are opening up large families of layered oxide topologies in thin film form, such as the *A*_2_*A*′_*n*−1_*B*_*n*_O_3*n*+1_ Ruddlesden–Popper (RP), *AA*′_*n*−1_*B*_*n*_O_3*n*+1_ Dion–Jacobson, (Bi_2_O_2_)(*A*_*n*−1_*B*_*n*_O_3*n*+1_) Aurivillius and (*AB*O_3_)_*m*_/(*A′B′*O_3_)_*n*_ perovskite superlattices^6^,^7^,^8^,^9^,^10^,^11^,^12^,^13^,^14^,^15^,^16^,^17^,^18^,^19^,^20^,^21^,^22^,^23^,^24^,^25^. The layer dimensions (subscripts *m* and *n*) of these materials along with strain and chemical ordering can be tuned to modify or activate new phenomena, such as ferroelectricity^26^,^27^, colossal-magnetoresistance^28^,^29^, ferromagnetism^30^,^31^, multiferroicity^32^, superconductivity^33^ and ionic conductivity^34^.

The key reason for such richness of phenomena in layered oxides versus simple perovskites is the presence of additional atomic plane inserts in a layered structure that introduce two-dimensionality as well as new physics at these interfaces. For example, atomic rumpling due to (SrO)_2_ rock salt layers in biaxially strained Sr_*n*+1_Ti_*n*_O_3*n*+1_ is predicted by density functional theory (DFT) to lead to a large number of competing ferroic states due to a breakdown of long range coherence of polarization between neighbouring perovskite blocks^35^. It was predicted and experimentally shown that by tuning the layer dimension (*n*) under biaxial tensile strain, ferroelectricity is induced. Biaxial tensile strained thin films for *n*=1–6 Sr_*n*+1_Ti_*n*_O_3*n*+1_ were realized in the form of thin films and were found to possess an impressive figure of merit (dielectric tunability divided by the dielectric loss) up to 125 GHz^26^; the ability of RP structures to form crystallographic shear planes, as opposed to point defects, was proposed to play a key role in lowering dielectric losses and enhancing this figure of merit. To understand the origins of such property enhancements, an atomic-level probing of the predicted phenomena of rock salt layer rumpling, decoherence between perovskite blocks and phase competition is critical.

In this work, using aberration-corrected high-resolution scanning transmission electron microscopy (HR-STEM) with sub-Å resolution, we image the atomic structure of a ferroelectric (FE) Sr_*n*+1_Ti_*n*_O_3*n*+1_ RP thin film. We show that the structure contains competing and coexisting FE distortions as predicted by DFT, as well as complex ordered polar regions at finite temperatures. In addition, we show that the presence of rock salt stacking faults introduces large structural distortions that lead to new low-symmetry phases not predicted by DFT. Taken together, this study reveals an exceptionally rich energy landscape in a RP structure that leads to a highly competitive ground state, a precursor to emergent phenomena and enhanced properties.

## Results

### DFT predictions of the strained Sr_*n*+1_Ti_*n*_O_3*n*+1_ RP phase

**Figure 1:**
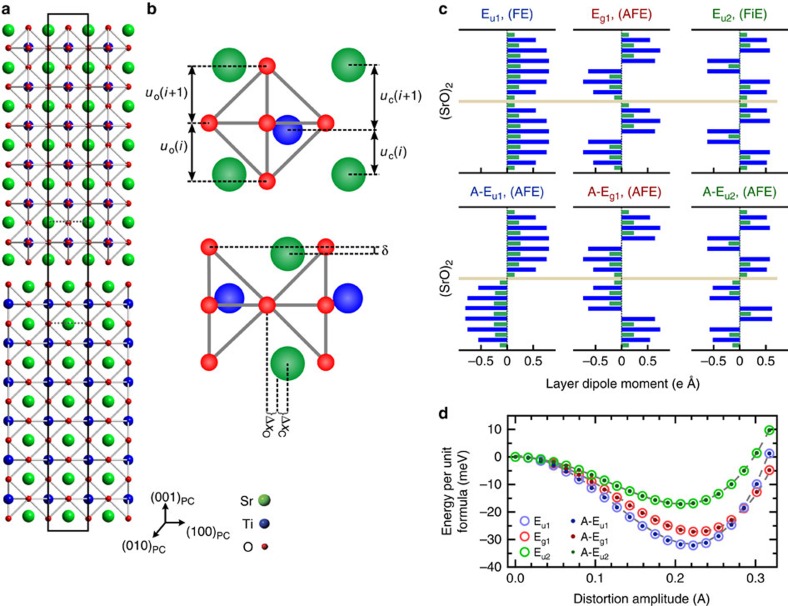
Atomic structure and diversity of FE phases of Sr_7_Ti_6_O_19_. (**a**) DFT-calculated paraelectric unit cell structure, black outline, of Sr_7_Ti_6_O_19_ with the rock salt layer (SrO)_2_ in the middle. (**b**) Enlarged view of the rock salt region showing the correlated rumpling *δ* and the cation and oxygen interplanar distances, *u*_C_ and *u*_O_, respectively due to the presence of the rock salt layer. The polar displacements of the cations Δ*x*_C_ and oxygen Δ*x*_O_ atoms away from their paraelectric state positions are shown. All distortions are exaggerated for clarity. (**c**) DFT-calculated layer dipole moment for the SrO (blue bars) and TiO_2_ (green bars) layers along the [100]_PC_ axis for the predicted FE, FiE and AFE unit cells. The unit cell net polarization for each nanoslab lies along one of the 〈110〉_PC_ directions. The three metastable phases, E_u1_, E_g1_, and E_u2_, arise from the unstable Γ-point phonons, and the A-E_u1_, A-E_g1_, and A-E_u2_ phases, are obtained by zone boundary (*k*_z_=*π*/*c*) phonons. (**d**) Calculated energy as a function of displacement (mode amplitude) for the various phonons. Note that the Γ-point modes are degenerate to those of the corresponding zone boundary instabilities.

We begin by describing the DFT-calculated atomic-level structural features of Sr_*n*+1_Ti_*n*_O_3*n*+1_ that are critical to the emergence of competing phases, and hence are the focus of the experiments that follow. The introduction of an extra SrO rock salt layer per perovskite block, Sr_*n+1*_Ti_*n*_O_3*n*+1_, makes the unit cell tetragonal and introduces two types of distortions as shown in Fig. 1a,b: (1) rumpling *δ*, which is a non-zero distance separating the cations and oxygen projected along [001]_PC_ (subscript PC for pseudocubic notation) within a SrO or TiO_2_ plane and (2) inhomogeneous interlayer separations *u*_C_ between cations and *u*_O_ between oxygens. These two distortions are correlated, since the rumpling *δ* causes the interlayer separations *u*_C_ and *u*_O_ to be different. Hence in the rest of this work, we experimentally measure interlayer separations and treat them as direct evidence for rumpling. These distortions are energetically favourable for both the polar and the paraelectric structures across all dimensionalities *n*, with biaxial strain having little influence (<15%) on the rumpling behaviour throughout the relevant strain range (0–1.7%). Figure 1c plots the layer-by-layer dipole moment in each atomic plane (001)_PC_ within a conventional cell projected along the [010]_PC_ direction for six different metastable phases. Of these, three stem from zone centre (Γ point) phonons which are labelled as E_u1_, E_g1_, and E_u2_ in Fig. 1c; these respectively correspond to FE, antiferroelectric (AFE) and ferrielectric (FiE) phases. There are three other unstable Γ-point phonons; however, the corresponding metastable phases could not be stabilized (see Supplementary Fig. 1). In addition, three zone boundary phonons (with *k*_z_=*π*/*c*), namely A-E_u1_, A-E_g1_ and A-E_u2_, can also be stabilized and result in nonpolar phases as shown in Fig. 1d. The net polarization along [001]_PC_ of the unit cell is zero for all the structures shown in Fig. 1; a typical calculation is shown in Supplementary Fig. 2.

Layer dimension (*n*) and strain play a key role in realizing FE phases in strontium titanate RP phases. The lowest energy symmetries of Sr_7_Ti_6_O_19_ (*n*=6) under a 1.7% tensile strain is predicted by DFT to be *Fmm*2 for the FE E_u1_ phase and *Cmcm* for the AFE A-E_u1_ phase as seen in Fig. 1c; the energy gains from these two instabilities are equal. Further degeneracies arise because adjacent perovskite blocks can have polarizations rotate into any of the four 〈110〉_PC_ directions, while retaining the projected polarization profiles shown in Fig. 1b. Given the close energy scales, it might be possible to observe the other metastable phases as well, especially when the structure is perturbed by finite temperature or extrinsic defects. The phases shown here are possibly the most obvious of an infinite set of ground and metastable states with different wavevectors, indicating that the energy landscape of Sr_*n*+1_Ti_*n*_O_3*n*+1_ RP under large tensile strain is extremely competitive.

Calculations show that at 0 K there are octahedral rotations in Sr_7_Ti_6_O_19_, in addition to the (anti-)polar distortions as discussed in Supplementary Note 1 and Supplementary Figs 3 and 4. Finite temperature prediction of the crystal structure is beyond the scope of this study. However, since the force constant matrix eigenvalue of the octahedral rotational instability (−0.30 eV Å^−2^) is almost an order of magnitude smaller than that of the FE mode (−2.7 eV Å^−2^), we expect that the onset of octahedral rotations occurs at a much lower temperature than the FE transition temperature of ∼240 K as described in Supplementary Note 2. As a result, it is very unlikely to see any signature of octahedral rotations in our room temperature (293 K) measurements, and this is consistent with our experimental observations. The .cif files for all the structures generated by the DFT study have been included in Supplementary Datasets 1–4, which can provide a reader with a full three-dimensional (3D) perspective on these structures.

### Aberration-corrected transmission electron microscopy

To test the above DFT predictions, aberration-corrected HR-STEM images were collected on 20 nm thick *n*=6 Sr_*n*+1_Ti_*n*_O_3*n*+1_ RP thin films grown on GdScO_3_ (101)_*Pnma*_ substrates. The films were grown by molecular beam epitaxy as described in Methods. The lattice mismatch between the film and substrate introduces a tensile 1.7% in-plane biaxial strain into the Sr_7_Ti_6_O_19_ thin film which has been shown to induce ferroelectricity in Sr_*n*+1_Ti_*n*_O_3*n*+1_ (ref. 26). Dielectric measurements indicate a Curie temperature *T*_c_∼240 K with a wide peak that spans over 200 K, as shown in Supplementary Fig. 5. STEM imaging was performed at 300 K (∼25 meV), which is ∼60 K above the average *T*_c_ but well within the transition region shown by the dielectric measurements. Also, as seen from the energy scales in Fig. 1c, a variety of phases become competitive at room temperature. High-angle annular dark field (HAADF) STEM images and X-ray diffraction *θ*–2*θ* scans of the Sr_7_Ti_6_O_19_ thin films show that the films are epitaxially grown, commensurately strained to the underlying substrate, and exhibit macroscopic *n*=6 layering as seen in Supplementary Figs 6 and 7.

### Atomic imaging of FE-like regions

**Figure 2:**
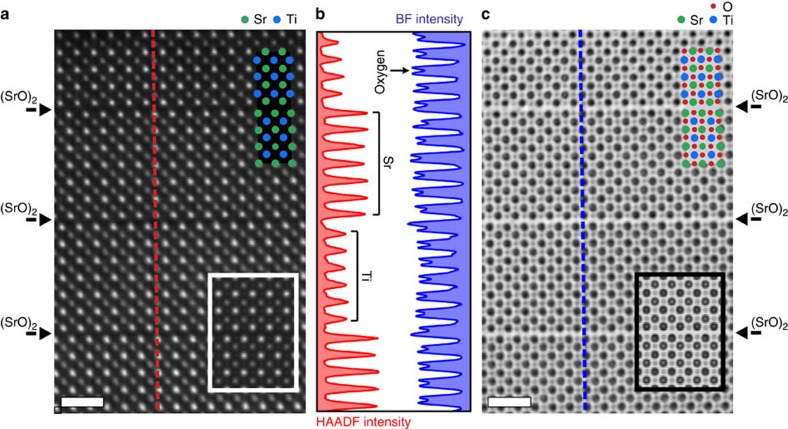
Atomic images of Sr_7_Ti_6_O_19_ thin film grown on GdScO_3_ (110)_*Pnma*_. (**a**) Drift-corrected high-resolution HAADF-STEM image of the (SrO)_2_ rock salt and perovskite blocks of the RP structure with the corresponding simulated HAADF image, in the white box, of the FE DFT structure from region #1 in the film. The strontium (filled green circles) and titanium (filled blue circles) atoms are shown for reference. (**b**) Intensity line profiles taken from the HAADF (red) and BF (blue) STEM images. The HAADF intensity shows the alternating ordering of the strontium and titanium atoms in neighbouring perovskite slabs while the BF intensity profile clearly shows that the oxygen atomic columns are resolved in the BF image. (**c**) Simultaneous BF image showing oxygen atomic columns along with a simulated BF image within the black box in the lower right. The oxygen atomic columns are highlighted by the superimposed filled red dots. Scale bar, 1 nm.

Figure 2 shows a representative high-resolution HAADF-STEM and bright field (BF) image taken along the [010]_PC_ direction, a region of the film free of stacking faults. Well-resolved individual atomic columns of strontium and titanium are clearly visible as white dots from the HAADF image in Fig. 2a. Simulated HAADF images using the FE DFT structure are laid over the experimental images and show excellent agreement with the observed HAADF-STEM images. The intensity line profile taken from the HAADF image across the rock salt layers is plotted in Fig. 2b, which shows uniform intensities for the strontium and titanium atoms across and in the neighbouring perovskite blocks, further confirming the RP structure. Figure 2c contains the simultaneously collected BF-STEM image, where the black dots represent the atomic columns. As with the HAADF image, the simulated BF image shows good agreement with the collected BF image. The oxygen atomic columns are clearly resolved in the line profile from the BF-STEM image.

**Figure 3:**
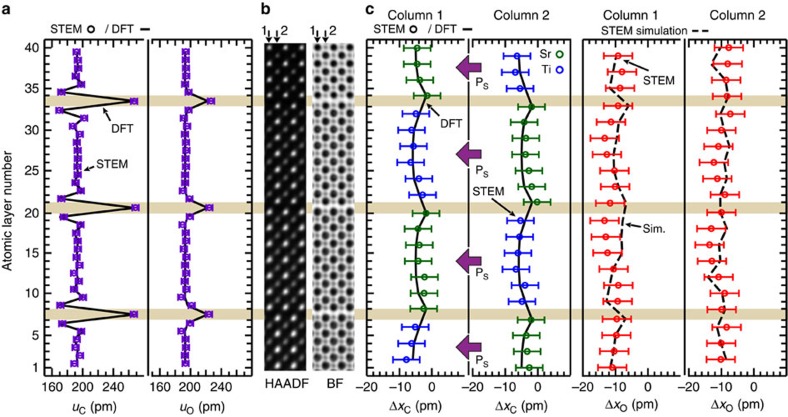
FE atomic columns in Sr_7_Ti_6_O_19_ film region #1. (**a**) Cation *u*_C_ and oxygen *u*_O_ interatomic spacing (purple open circle) extracted from the drift-corrected HAADF and BF STEM images superimposed on the DFT calculated values (solid grey line). The characteristic decrease in interplanar spacing in *u*_C_ and its absence in *u*_O_ adjacent to the rock salt layers (horizontal beige colour rectangles across the panels) indicates rumpling in the atomic structure due to the rock salt layer. Exceptional agreement between experiments and DFT is seen. (**b**) Average experimental HAADF and BF STEM slices from region #1 of the sample. The slices are repeated for clarity. (**c**) Cation Δ*x*_C_ (blue and green open circles) and oxygen Δ*x*_O_ (red open circles) displacements along the [100]_PC_ taken from the drift-corrected HAADF and BF images along with the superimposed FE DFT calculated positions (solid black line) and FE DFT simulated oxygen positions (dashed black lines). The simulated oxygen positions were performed with a 1.25 mrad tilt about the [001]_PC_ axis (the simulation with the best agreement to experiment). The error bars are taken to be the root mean s.e. from the positions of a best fit lattice determined by CV.

To quantify the atomic positions within the RP structure, images from several areas of the film along the [100]_PC_ were collected (Supplementary Figs 8–11). Analysis of the structures was performed in regions free of stacking faults by constructing average slices one unit cell wide along the growth direction. Of the above discussed areas, three were found to possess in-plane distortions characteristic of the FE and AFE phase, and are hence labelled regions #1–3. Figure 3a shows a direct comparison of the measured cation *u*_C_ and oxygen *u*_O_ interplanar spacing for the average atomic slices from region #1 with the DFT predicted values. As seen, there is an exceptional match with the DFT-calculated *u*_C_ and *u*_O_ values, including the characteristic decrease in *u*_C_ next to the rock salt layer. This is all the more remarkable considering it is a direct comparison between image simulations of the DFT structure and STEM experiments with the only post-processing of the images being drift correction, as detailed in Methods. To show that such an agreement is neither accidental nor limited to the FE structure, the *u*_C_ and *u*_O_ values were measured in an *n*=4 Sr_*n*+1_Ti_*n*_O_3*n*+1_ film grown on GdScO_3_ (101)_*Pnma*_ and is shown in Supplementary Fig. 12. These results prove the DFT prediction of atomic rumpling in both the paraelectric and FE structures in all its intricate detail.

Next we investigate the polarization state of an 8 × 8 nm^2^ area of the film with the well-defined RP structure in region #1. Figure 3b,c shows a direct comparison of the measured cation displacements, Δ*x*_C_ and oxygen displacements, Δ*x*_O_, with the DFT predictions; cationic atomic column 1 and the adjacent column 2 show excellent agreement with DFT as shown in Fig. 3c. The oxygen positions also match the experiments quite well as shown in Fig. 3c, after accounting for a 1.25 mrad experimental tilt in the simulations (see Supplementary Fig. 13). The simulations show that unlike the oxygen atomic columns, sample tilt has minimal impact on the cation positions. Based upon the observed Δ*x*_C_ values, we find that this region of the sample is FE-like. Considering that the DFT predictions are at 0 K and the measurements are near 300 K, this suggests that localized atomic columns in the film remarkably retain cationic displacements similar to predicted low-temperature phases. DFT further suggests that for the same polarization projection profile along [00]_PC_, as seen experimentally, multiple nearly degenerate states could exist. For example, the polarization in each perovskite block might be in the same 〈110〉_PC_ direction, or it could rotate between the four equivalent 〈110〉_PC_ directions between the neighbouring perovskite blocks such that the pseudo-cubic polarization projection remains along [00]_PC_. The energy difference between these multiple degenerate competing states is essentially zero according to our DFT calculations. A similar analysis of region #3 (Supplementary Fig. 14) shows that it is also FE-like similar to region #1. These results, taken together, suggest that localized atomic columns in the film are FE as predicted by the DFT, even at a temperature slightly above the nominal *T*_c_ measured from the macroscopic dielectric data. To understand the 3D nature of this ferroic phase, we have also analysed images from the [110]_PC_ zone axis, as shown in Supplementary Fig. 15. The results show that, in this region, while the polar displacements of the Ti cations are larger than that of the Sr atoms, the cation displacements are consistent with the expected projected displacements for Γ_5−_irreducible representation from the DFT-calculated FE structure.

### Atomic imaging of unknown phases

**Figure 4:**
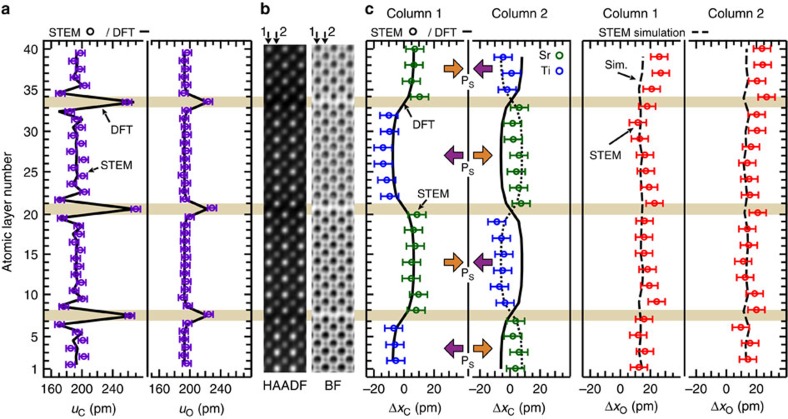
Unknown AFE-like atomic columns in Sr_7_Ti_6_O_19_ film region #2. (**a**) Cation *u*_C_ and oxygen *u*_O_ interatomic spacing (purple open circles) extracted from constructed drift-corrected HAADF and BF STEM images superimposed on the DFT calculated values (black lines). The characteristic decrease in interplanar spacing in *u*_C_ and absence in *u*_O_ adjacent to the rock salt layers indicates the presence of rumpling at the rock salt layer; exceptional agreement between experiments and DFT is seen. (**b**) Constructed average HAADF and BF STEM slices, which are repeated for clarity. (**c**) Cation Δ*x*_C_ (blue and green open circles) and oxygen Δ*x*_O_ (red open circles) displacements along the [100]_PC_ direction measured from the drift-corrected HAADF and BF images. The AFE DFT calculated value (solid black lines) and the AFE STEM simulated Δ*x*_O_ values (dashed light blue line) are superimposed to show agreement between simulation and experiments. Included in column 2 is the *π* phase shifted cation positions (dotted black line) for comparison with the STEM data. The simulated Δ*x*_O_ values (dashed black lines) were done with a 1.25 mrad sample tilt around the [001]_PC_ axis. The error bars are taken to be the root mean s.e. from the positions of a best fit lattice determined by CV.

To confirm the presence of multiple degenerate states, we compare the above results with those from a second area of the sample, region #2. HAADF and BF images were collected from these areas. As with region #1, the presence of rumpling within the RP structure is clearly confirmed over an 8 × 6 nm^2^ region in Fig. 4a by the measured *u*_C_ and *u*_O_ values in sample region #2. Note again the exceptional agreement between DFT and experiments for the rocksalt rumpling. The richness of ferroic phases is illustrated by analysing two adjacent columns of atoms in this region as shown in Fig. 4b. Figure 4c depicts that Δ*x*_C_ values measured in column 1 closely match the expected displacement profile for the AFE-like structure with polarization in the ±[100]_PC_ directions. The AFE-like atomic column is consistent with the expected projected displacements for the M4-irreducible representation from DFT. Surprisingly, however, the Δ*x*_C_ values in adjacent column 2 also exhibits an AFE-like distortion, except it is reversed by a phase of *π* with respect to column 1. This alternation of the AFE-like antiphase distortion over adjacent columns remarkably extends over ∼8 nm in this region, indicating that this is an ordered new phase which is AFE along both the transverse and the longitudinal directions. While such a structure would be electrostatically unfavourable in an insulating state, it may be stabilized at finite temperatures if finite conductivity exists, for example, due to point defects. It is also likely that at room temperature (25 meV), which is slightly above the FE transition, the relative enthalpies of the different phases are similar and hence many such phases, some unknown, can coexist in an intricate atomic scale mixture. Such unknown ordered phases close to phase transition, not previously predicted by DFT, have been seen in other FEs^36^.

**Figure 5:**
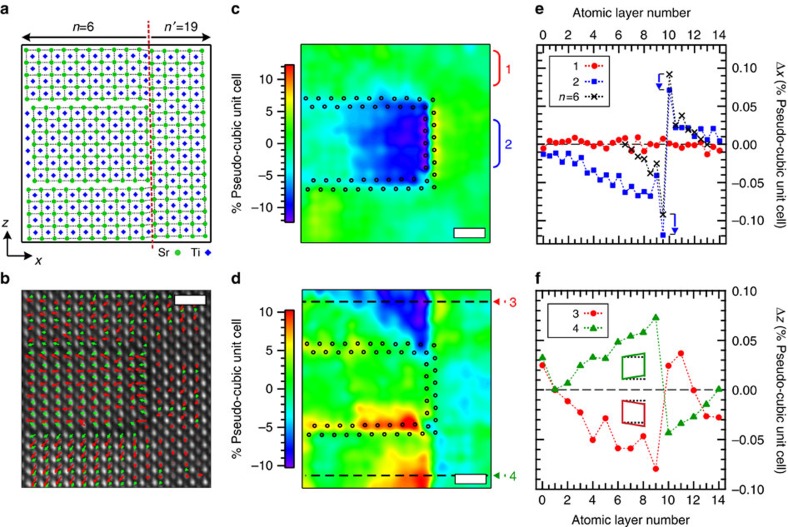
Distortion of new polar phases near a (SrO)_2_ stacking fault in Sr_7_Ti_6_O_19_. (**a**) Model structure of a stacking fault for the *n*=6 Sr_*n*+1_Ti_*n*_O_3*n*+1_ RP structure. To the left of the stacking fault (red dashed line) is the *n*=6 structure while to the right is a perovskite slab of the *n*′=19 structure. The strontium and titanium atoms are given by green circles and blue diamonds, respectively. (**b**) Drift-corrected HAADF image of a stacking fault. Superimposed arrows qualitatively show the SrO and TiO_2_ layer dipole moment near the stacking fault (**c**,**d**) Colour map of difference between atomic positions of the model and HAADF-STEM derived cation positions perpendicular Δ*x* (panel **c**) and parallel Δ*z* (panel **d**) to the stacking fault. The Δ*x* and Δ*z* maps show that the accommodation of the lattice mismatch between the *n*=6 and *n*′=19 structure near the stacking fault is predominantly contained within the *n*=6 region. The (SrO)_2_ rock salt layers are indicated by the black open circles. (**e**) Line profile of Δ*x* (filled red circles) taken along the horizontal dashed line labelled (1) in panel (**c**) and its comparison to an ideal *n*=6 structure across the rock salt layer (open blue squares) highlight an ∼10 pm shift of the rock salt leading to a compressive strain in the *n*=6 layer. (**f**) Line profile taken along the dashed horizontal line labelled (2) in panel (**d**) from selected region #2 in (**d**) showing a monoclinic-like distortion (inset schematic) of the SrTiO_3_ pseudo-cubic unit cell across the stacking fault. Scale bar, 1 nm.

In addition to the rock salt layers perpendicular to the [001]_PC_ *c* axis that define the RP structure, the films also possess rock salt stacking fault planes that run parallel to the [001]_PC_ direction. It was suggested by Lee *et al*. that the preference of an RP structure to incorporate such defects (as opposed to point defects) might be the key to achieving the observed low dielectric losses^26^. In the simplest case, the stacking fault interrupts and connects two neighbouring (SrO)_2_ layers which results in an *n*′=3*n*+1 RP region on one side of the stacking fault and a conventional RP layer with dimensionality *n* on the other side (see Fig. 5a). For an *n*=6 RP, this would result in an *n*′=19 structure on the opposite side of the stacking fault. Because each added (SrO)_2_ rock salt layer added to an RP has a larger lattice parameter than a SrTiO_3_ perovskite block, the *c*-axis lattice constant of the *n*=6 RP structure (which contains 12 perovskite blocks and one (SrO)_2_ rock salt layer) is larger than the neighbouring 13 perovskite blocks in the *n*′ RP layer. Based on DFT calculations of 1.7% biaxial strained SrTiO_3_, this difference in lattice constant is expected to be roughly 1.3%. By comparing a reference *n*=6 region to the *n*′=19 region in drift-corrected HAADF images (see a larger region in Supplementary Fig. 16) we find this value to be ∼2.0%. Using this STEM-derived mismatch strain along with the DFT calculated *n*=6 RP and SrTiO_3_ structure, we constructed a simple atomic model of the stacking fault. This model contains the expected rumpling in the *n*=6 structure along [001]_PC,_ but has a constant in-plane atomic spacing across the stacking fault along [100]_PC_ defined by reference region, as explained in Supplementary Fig. 16. By employing a constant [100]_PC_ lattice spacing, lattice distortions due to the presence of the stacking fault are highlighted.

To qualify the local inhomogeneity introduced by the stacking fault, a layer-by-layer dipole moment within the structure, (Fig. 5b), was constructed using the oxygen and cation positions obtained from the HAADF and BF images, respectively, shown in Supplementary Fig. 16. From this map it is clear that the SrTiO_3_ pseudo-cubic structure is not consistent within the *n*=6 or *n*′=19 regions. For example, there appears to be a large out-of-plane polarization in the bottom perovskite slab in the *n*=6 region, while the polarization is minimal in the *n*′=19 region. To further understand these structural differences, we looked at how the STEM-derived atomic structure deviates from the model along directions perpendicular (Δ*x*) and parallel (Δ*z*) to the stacking faults, highlighted by the colour maps in Fig. 5c,d. Interestingly, these maps shows that the transition region accommodating the out-of-plane lattice mismatch of the *n*=6 and *n*′=19 regions predominantly occurs within the *n*=6 region. Further inspection of the Δ*x* values in Fig. 5e reveals that there is an in-plane compressive strain within only the centre *n*=6 perovskite layer that extends up to eight unit cells away from the stacking fault before the lattice parameter returns to the epitaxially strained value. In addition, we find in Fig. 5e that the stacking fault is shifted Δ*x*∼10 pm to the left towards the *n*=6 region from its expected position. This asymmetric shift helps to explain why a larger distance is required for the *n*=6 region to return to the epitaxial strain value as compared with the *n*′=19 region. Unlike Δ*x*, we find that the large values of Δ*z* occur in the top and bottom perovskite layers on the *n*=6 side as seen in Fig. 5c. Analysis of these regions indicates that the lattice mismatch of the *n*=6 and *n*′=19 structure introduces shear distortions into the SrTiO_3_ pseudo-cubic unit cells, as shown by the line profile and the inset schematic in Fig. 5f. As with Δ*x*, we find that the distortion in Δ*z* propagates further into the *n*=6 region than into the *n*′=19 region. We note that the opposing dipoles created at the rocksalt interface are electrostatically unfavourable. However, the coordination environment of the ions on the interface are significantly different from their counterparts within the perovskite layers. For example, each oxygen ion within the perovskite blocks are bonded with two titanium ions, whereas the oxygens on the interface (double rocksalt) layers are bonded with only one titanium. The resulting asymmetry in the chemical bonding is a driving force for displacement of ions on the interface. We note that even the high-symmetry (paraelectric) structure of the RP compounds have considerable rumpling on the interface layers, which create large dipoles that point away from the interface. Thus we observe that new metastable phases are stabilized in the proximity of a vertical rock salt stacking fault, with complex polarization profiles that include out-of-plane polarization, which are not predicted by DFT.

In-depth analysis of the atomic structure and DFT calculations show a reasonable agreement for regions #1 and #3 where there are no extrinsic defects. In region #2, we see an unknown ordered AFE-like phase, while in the vicinity of stacking faults (Fig. 5), the lattice distorts and new complex polar phases arise. Our overall analysis of these and other different regions within the film suggest no systematic variation in trends, where a random mixture of FE-like, AFE-like, PE-like and complex polar phases coexist. This is expected at room temperature, slightly above the *T*_c_, where competing degenerate ferroic phases are expected to occur as an intricate atomic scale mixture.

## Discussion

The original prediction of strain-induced ferroelectricity in Sr_*n*+1_Ti_*n*_O_3*n*+1_ by Birol *et al*.^35^ concludes as follows: ‘All of this suggests that there are an infinite number of degenerate states involving uncorrelated atomic-scale polar regions. We surmise that the ground state may be a form of relaxor ferroelectricity without disorder'. This work provides the first atomic-scale evidence for such a rich landscape of competing phases in a layered oxide. By a direct comparison between atomic-scale imaging and DFT, this work shows that the RP thin films under large tensile strain are a host to a large number of competing phases at room temperature that can possess FE, AFE, FiE, paraelectric and other complex phases. Layered oxides such as the RP structures studied here provide a natural mechanism to decouple adjacent perovskite blocks through rumpling across a rock salt layer that is directly imaged in this work and shows exceptional agreement with DFT predictions.

A combination of a highly degenerate energy landscape consisting of many competing phases, combined with decoupled two-dimensional polar blocks separated by rock salt layers could be a basis for the design of new classes of digital relaxors and electrocaloric materials. In relaxors, the availability of a large configurational space for polarization to point in, allows for enhanced electromechanical and dielectric response^37^. Similarly, the temperature change achieved in an electrocaloric material by undergoing an order–disorder transition is proportional to the log of the number of the available configurational degrees of freedom. Thus, new design paradigms are possible in a layered structure wherein phase competition is exploited towards increasing nanoscale entropy and to achieve large property enhancements. While the conventional approach of achieving phase competition in oxides is largely through inducing chemical disorder, this study suggests alternate paths by tuning a layered structure with topology and strain knobs. The quantitative sub-Å imaging and DFT study presented here provides a template for the materials-by-design paradigm by interfacing atomic-level theory and atomic-level experiments in a tight feedback loop.

## Methods

### Scanning transmission electron microscopy

Aberration-corrected HR-STEM images along (100)_PC_ were collected using the TEAM 0.5 microscope at the National Center for Electron Microscopy operating at 300 kV with a probe illumination angle of 17 mrad. Information regarding the cation and oxygen atomic positions were captured with simultaneous HAADF and BF detectors, respectively. The HAADF detector had a collection angle of 52–253 mrad while the BF detector had a collection angle of 0–12 mrad. Images for (100)_PC_ were collected with a defocus of ∼−100 Å. This negative defocus was used to achieve the optical contrast for interpreting the oxygen atomic columns, while only slightly reducing the sharpness of the HAADF images. The (110)_PC_ images were collected on an aberration-corrected HR-STEM at Penn State University operating at 300 kV with a probe illumination angle of 28 mrad. HAADF and ABF-like images were obtained with collection angles of 42–244 mrad and 9–51 mrad, respectively. At each sample location, images were taken with the STEM fast scan direction set to 0° and 90° with respect to the substrate interface direction. These image pairs were then drift corrected^38^, after which the images were superimposed.

### STEM analysis and simulations

Analysis of the STEM images was performed using custom-written MATLAB codes. The sub-pixel resolution of the cation positions were determined by fitting a seven parameter 2D elliptical Gaussian profile (to account for any ellipticity in the intensity distribution) to the HAADF intensity distribution. Meanwhile, the oxygen atom positions were determined by a multipeak seven parameter 2D elliptical Gaussian fit of the simultaneously collected BF images; the fit was used to account for the cation atomic column intensity tails. The BF and HAADF slices analysed in Figs 3 and 4 were generated by first segmenting the BF and HAADF images based upon a grid 1 PC UC wide along the [100]_PC_ direction and then taking the average intensity distribution of these segments.

HAADF- and BF-STEM image simulation at 300 kV was performed using the multislice method implemented in MATLAB code, following the methods given by Kirkland^39^. The simulated angular detection angle for HAADF and BF were 53–100 mrad and 0–12 mrad, respectively, with a maximum scattering angle of 5.08 Å^−1^. Simulations were performed with a probe spacing of 0.248 Å (below the measured HR-STEM image information limit of 0.7 Å) and an illumination semi-angle of 17.1 mrad for 16 different frozen phonon configurations which were averaged to generate the HAADF and BF images. Simulations were performed for different values of defocus, foil thickness and sample tilt which were compared with the STEM data to ensure reliable interpretation. Comparison with the STEM data reveals a tilt angle about the [001]_PC_ axis of 1.25 mrad.

Within the analysed area of the HAADF and BF images, we estimated the precision of the atomic positions as the root mean squared deviation (r.m.s.d.) from the site positions of a best fit lattice (Supplementary Fig. 17). To determine where the region of the sample was closest to the representative structure, we used a cross-validation (CV) approach. The CV procedure is as follows. First a subset of atomic positions was selected by identifying all of the atoms within a predefined fitting radius. Next, a best fit lattice was calculated by randomly selecting a set consisting of half of these atomic positions which were used to predict the location of the remaining half. The CV score is set equal to the r.m.s.d. of these predicted sites. This procedure was repeated over one thousand times, with each using a new randomly generated subset. This compilation of scores was then used to calculate the mean CV score for a particular fitting radius. The mean CV score procedure was then repeated for different fitting radii to determine the optimal number of sites that should be included in a linear best-fit lattice such that the lattice is not under-fit nor over-fit. This ideal fitting condition is met when the CV score reaches a minimum. At this CV minimum, the calculated RMS error represents the upper estimate limit of precision. Because the cations and oxygen atom positions are derived from the HAADF and BF images, respectively, this CV analysis was done on both the HAADF and BF images.

### Summary of DFT methods

First-principles calculations are performed with Projector Augmented Waves^40^,^41^ as implemented in the Vienna Ab initio stimulation package (VASP)^42^,^43^. The potentials have the valence electron configurations 4*s*^2^4*p*^6^5*s*^2^ for Sr, 3*p*^6^4*s*^2^3*d*^4^ for Ti and 2*s*^2^2*p*^4^ for the O ions. Spin–orbit interaction is ignored, and PBEsol exchange correlation functional, which gives good structural parameters for noncorrelated oxides, is used^44^. A *k*-point grid that has eight points along the in-plane lattice vectors are used. The energy cutoff for the plane waves is chosen to be 500 eV, which is larger than the suggested value for the VASP potentials, and provides good convergence in these systems. To identify lattice instabilities, the dynamical matrices are built using the frozen phonons method (the direct approach). Relaxations of lattice structures to obtain the optimized structures are performed using the biaxial boundary conditions where the in-plane lattice constants are fixed, and the out-of-plane lattice parameter as well as the internal atomic coordinates are relaxed. These relaxations are performed keeping the space group fixed, which is *I*4/*mmm* (#139) for the paraelectric structure, and *C*2/*m* (#12) and *Fmm*2 (#42) for the structures corresponding to E_g_ and E_u_ distortions respectively. The stability of the metastable structures are checked against Γ point instabilities by repeating phonon calculations with the relaxed structures.

To calculate the layer-by-layer polarization, we used the Born effective charges (calculated in the paraelectric structure using density functional perturbation theory) and finite displacements. While this introduces an extra approximation that the Born Effective Charges do not change between the FE and paraelectric phases, this error is well known to be usually small in these systems compared with other systematic errors present in DFT calculations.

The Sc atoms in the GdScO_3_ substrate form a tetragonal surface layer rather than a square one. The lattice constants of this layer are 3.964 and 3.970 Å respectively, which corresponds to a 0.15% anisotropy. To verify that this small anisotropy does not lead to an important difference, we repeated our DFT calculations to relax the atoms with the boundary condition that the in-plane lattice constants are different by this amount. We could not find any physically meaningful difference in the energies of different phases, which shows that it is safe to ignore the anisotropy of the substrate.

At various points in this study, we made extensive use of the Isotropy Software Package (http://stokes.byu.edu/iso/isotropy.php) and the Bilbao Crystallographic Server^45^,^46^. Visualization of crystal structures and calculation of certain properties have been achieved using VESTA^47^.

### Data availability

The data that support the findings of this study are available from the corresponding author upon request.

## Additional information

**How to cite this article:** Stone, G. *et al*. Atomic scale imaging of competing polar states in a Ruddlesden–Popper layered oxide. *Nat. Commun.* 7:12572 doi: 10.1038/ncomms12572 (2016).

## Supplementary Material

Supplementary Information Supplementary Figures 1-17, Supplementary Notes 1-2 and Supplementary References
