# Supplementary material for: Atomic scale imaging of competing polar states in a Ruddlesden–Popper layered oxide
Source: Nat Commun. 2016 Aug 31;7:12572. doi: 10.1038/ncomms12572 (PMC5013660; doi:10.1038/ncomms12572)
Supplement: Supplementary Information — Supplementary Figures 1-17, Supplementary Notes 1-2 and Supplementary References [file ncomms12572-s1.pdf]

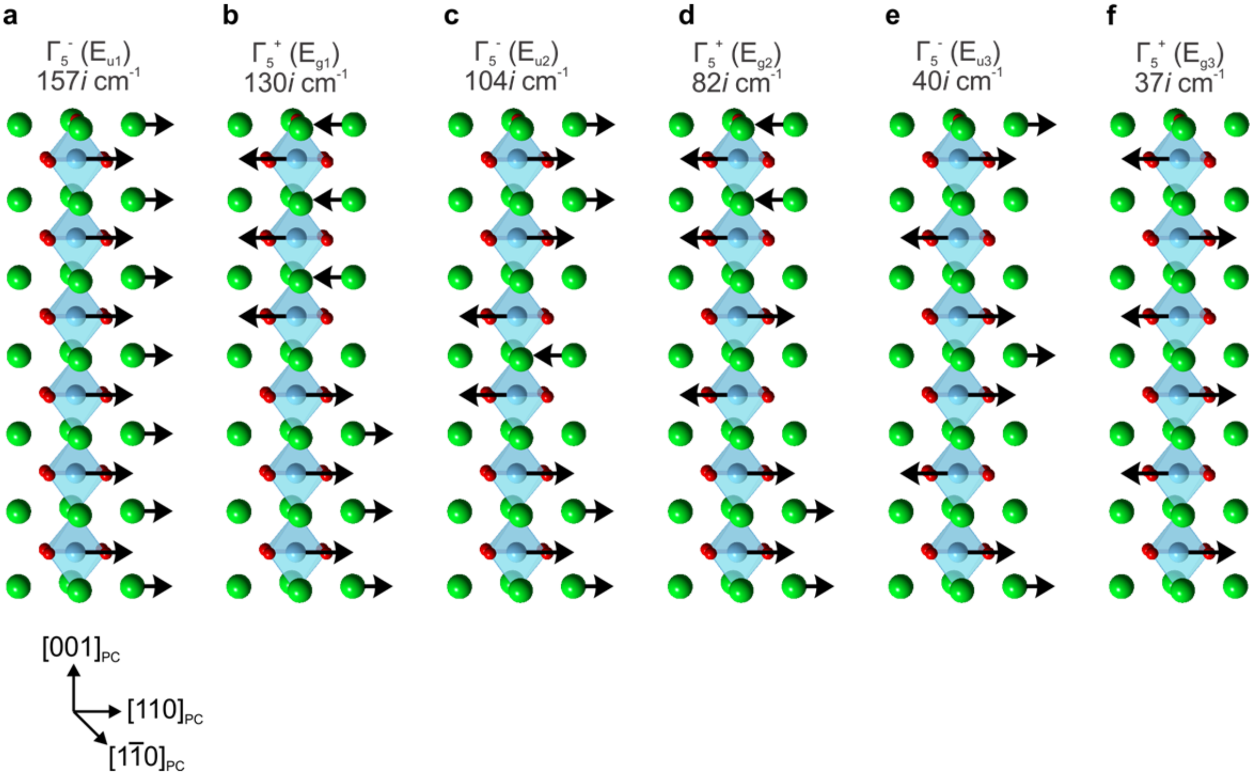

Supplementary Figure 1 | Density Functional Theory (DFT) calculated unstable Γ-point phonon modes in $Sr_7Ti_6O_{19}$. Qualitative displacement of the cations away from the parent paraelectric structure (black arrows) for six phonon modes at the gamma point predicted to be unstable by DFT calculations. The oxygen atomic columns in each layer displace in the opposite direction to the cations (not shown). Calculations are performed for $Sr_7Ti_6O_{19}$ with 1.7% tensile strain. For clarity, only one perovskite block is shown. The first three of these modes (**a**)-(**c**) E_{u1}, E_{g1}, and E_{u2} lead to (meta-)stable phases. However, the other three unstable modes (**d**)-(**f**) E_{g2}, E_{u3}, and E_{g3} do *not* lead to a stable phase. In other words, they correspond to a saddle point in the energy landscape rather than a local minimum. The reason, partly, is that they transform as the same irreducible representation as the other unstable modes that can further lower the energy without changing the symmetry of the crystal structure.

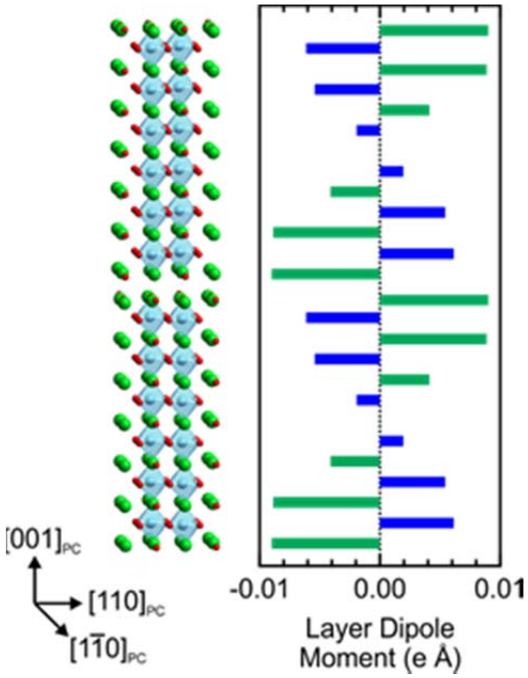

Supplementary Figure 2 | Density Functional Theory (DFT) calculated out-of-plane polarization in ferroelectric $Sr_7Ti_6O_{19}$. Layer-by-layer out-of-plane $[001]_{PC}$ polarization arising from ferroelectric displacements away from the paraelectric positions. The unit cell of $Sr_7Ti_6O_{19}$ is included for reference. A nearly identical profile is also seen for the other ferroic phases presented in this work. On the interface (double rock salt) layers, there are tail-to-tail dipoles which are electrostatically unfavorable, but are formed in order to optimize the local bonding environment of the ions.[1]

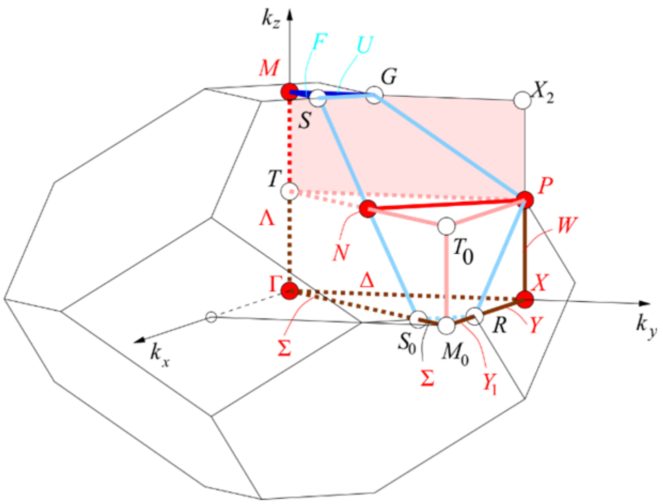

Supplementary Figure 3 | High symmetry wavevectors of the space group I4/mmm. The cell in the figure is the first Brillouin zone of the high symmetry (paraelectric) structure. Note that the x and y axes are rotated by 45 degrees from the tetragonal axes of the conventional cell, and the X point has the k-vector (π/a, π/a, 0) in the conventional basis. (Image from the Bilbao Crystallographic Server)

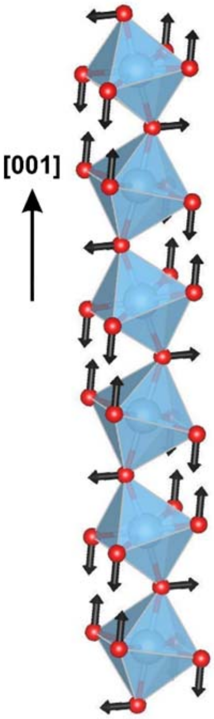

Supplementary Figure 4 | Oxygen Octahedral Rotational Instability in $Sr_7Ti_6O_{19}$. Schematic of the strongest rotational instability of $Sr_7Ti_6O_{19}$ at the X point (Glazer notation of $a^-a^-c^0/a^-a^-c^0$), shown for one perovskite layer. This instability involves 99% oxygen and ~1% Sr displacements. As discussed in the text, the force constants matrix eigenvalue of this mode (-0.30 eV Å^{-2}) is almost an order of magnitude smaller than that of the FE mode. Hence, we expect that the onset of octahedral rotations occur at a much lower temperature than the ferroelectric transition temperature. As a result, it is very unlikely to see any signature of octahedral rotations in our room temperature (293 K) measurements.

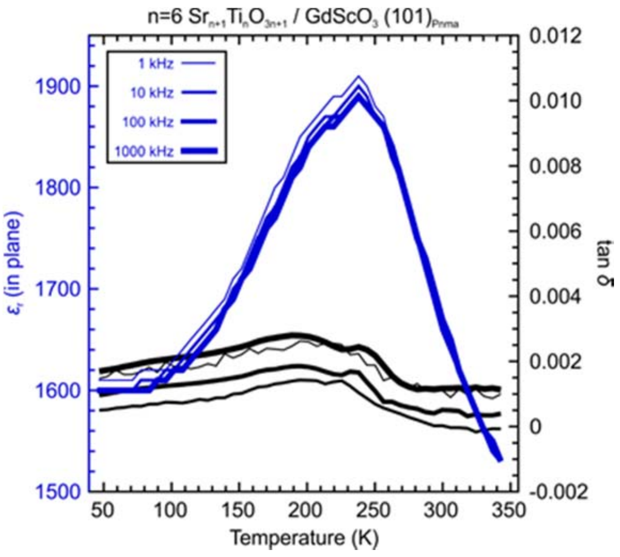

Supplementary Figure 5 | Dielectric and ferroelectric properties of $Sr_7Ti_6O_{19}$. Dielectric constant, ε_r, (in blue) and dielectric tangent losses, *tan δ*, (in black) for *n*=6, $Sr_7Ti_6O_{19}$, as a function of frequency and temperature. The peak in ε_r corresponds to a ferroelectric transition at ~240 K.

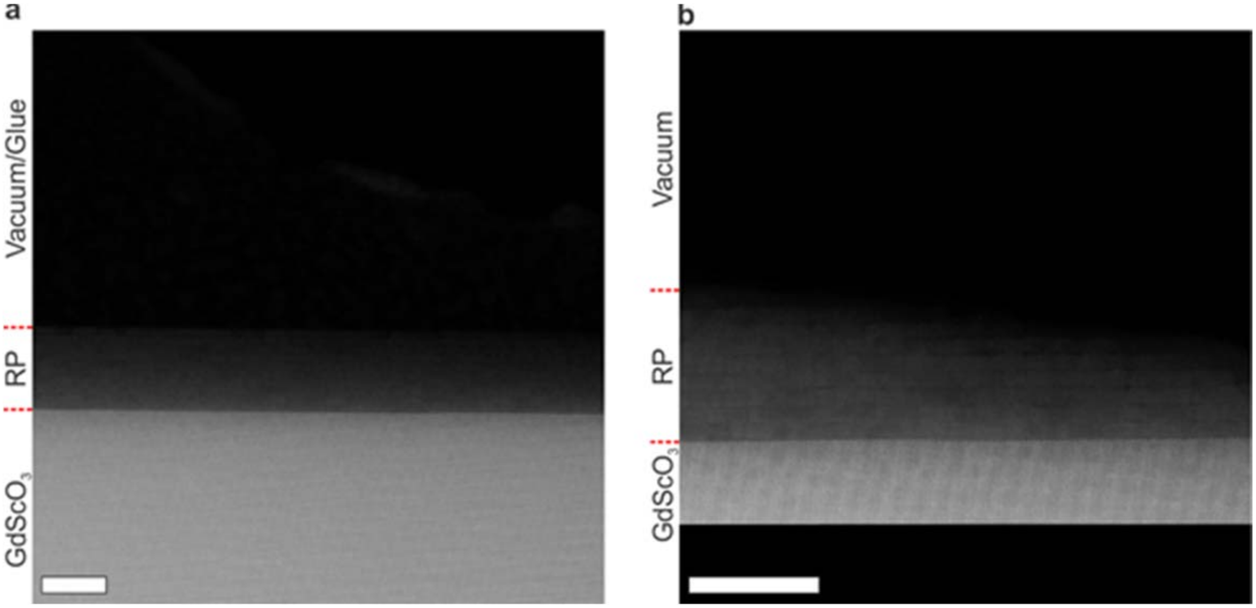

Supplementary Figure 6 | High Angle Annular Dark Field (HAADF) images of the $Sr_7Ti_6O_{19}$ thin film on $GdScO_3$ (101)$_{Pnma}$. HAADF images taken from two different areas of the thin film showing high quality, epitaxial growth, and the absence of threading dislocations. Scale bar, 20 nm.

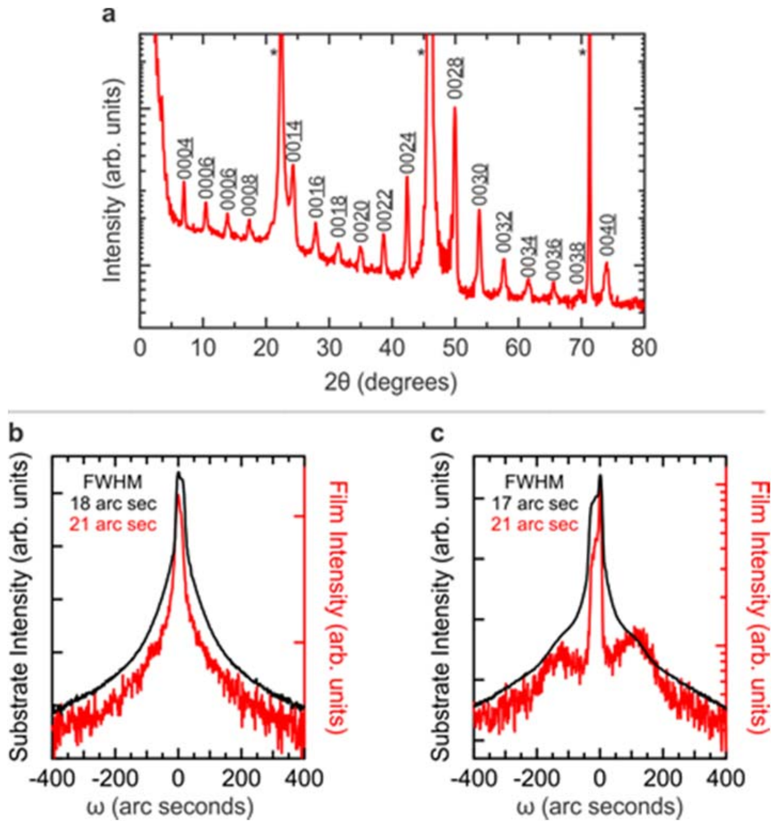

Supplementary Figure 7 | X-ray Diffraction (XRD) characterization epitaxial $Sr_7Ti_6O_{19}$ films grown on $GdScO_3$ (101)$_{Pnma}$. (**a**) θ-2θ XRD scan. Substrate peaks are labeled with an asterisk. (**b-c**) XRD rocking curves of the (00<u>26</u>) peak.

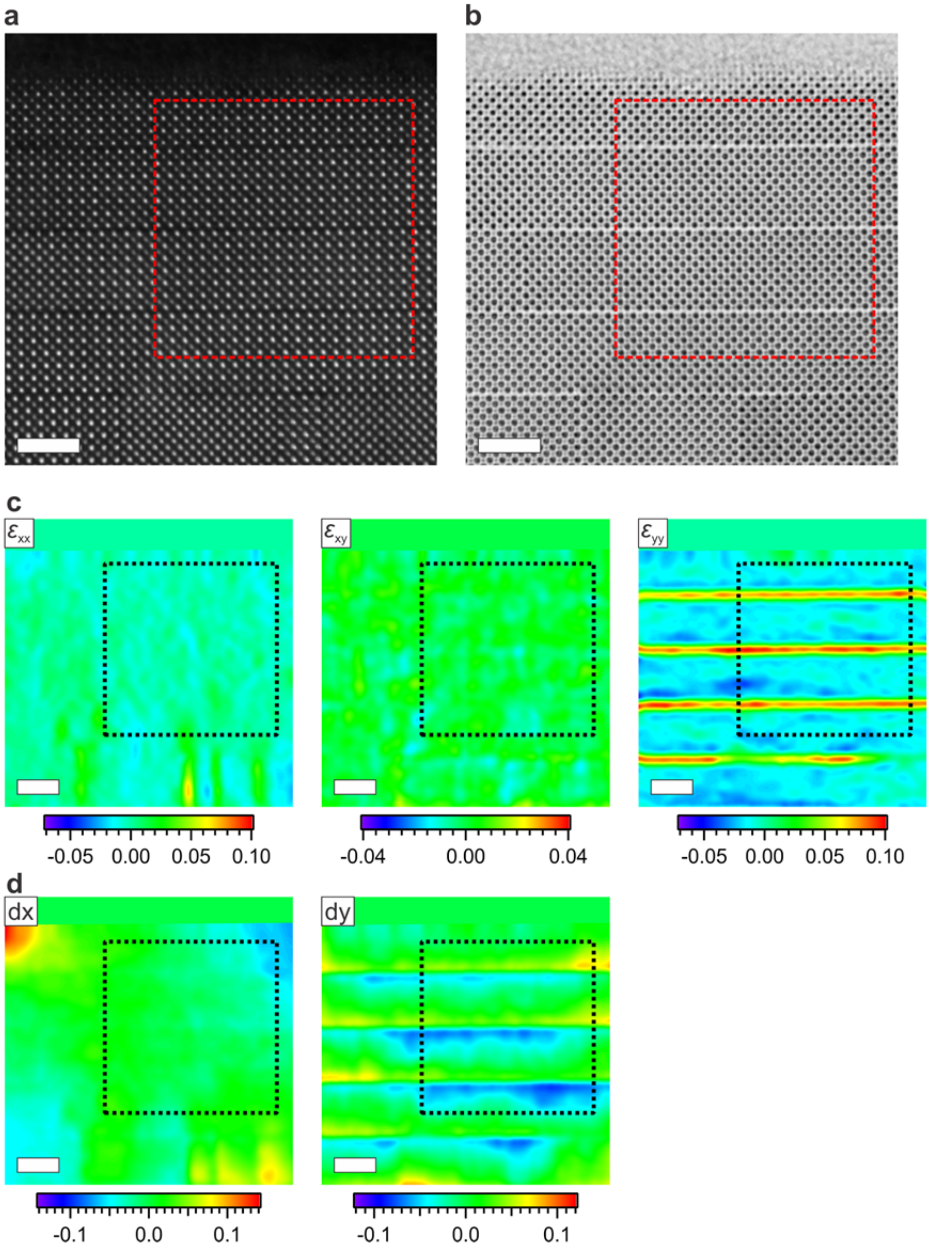

Supplementary Figure 8 | HAADF, BF, and strain maps of region #1 in the $Sr_7Ti_6O_{19}$ film. Drift corrected (**a**) HAADF and (**b**) BF images with the area of analysis highlighted by the dashed red box. (**c**) The ε_{xx}, ε_{xy}, and ε_{yy} strain maps with respect to the pseudo-cubic perovskite structure of the analysis area. The strain maps show that there are no long range structural ordering beyond the expected Ruddlesden-Popper structure in the area of analysis. (**d**) Color scale maps representing the deviation of the atomic columns from an overlaid cubic grid (dx and dy) calculated using all of the atomic columns within the HAADF image. Scale bar is 2 nm.

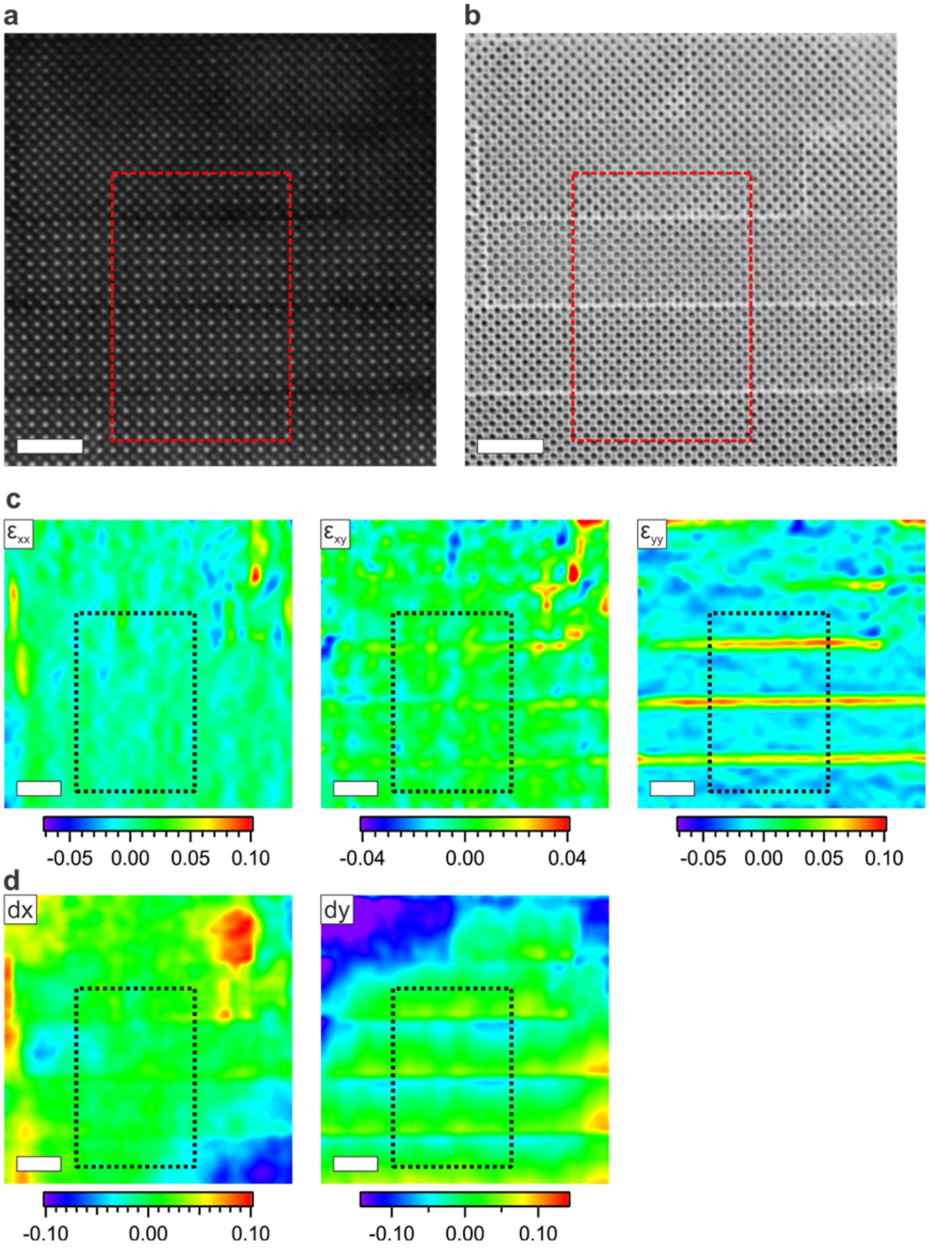

Supplementary Figure 9 | HAADF, BF, and strain maps of region # 2 in the $Sr_7Ti_6O_{19}$ film. Drift corrected (**a**) HAADF and (**b**) BF images with the area of analysis highlighted by the dashed red box. (**c**) The ε_{xx}, ε_{xy}, and ε_{yy} strain maps with respect to the pseudo-cubic perovskite structure of the analysis area. The strain maps show that there are no long range structural ordering beyond the expected Ruddlesden-Popper structure in the area of analysis. Ion and electron beam damage introduced to the structure near the rock salt layers is visible in the top and top right part of the images. (**d**) Color scale maps representing

the deviation of the atomic columns (dx and dy) from an overlaid cubic grid calculated using all of the atomic columns within the HAADF image. Scale bar is 2 nm.

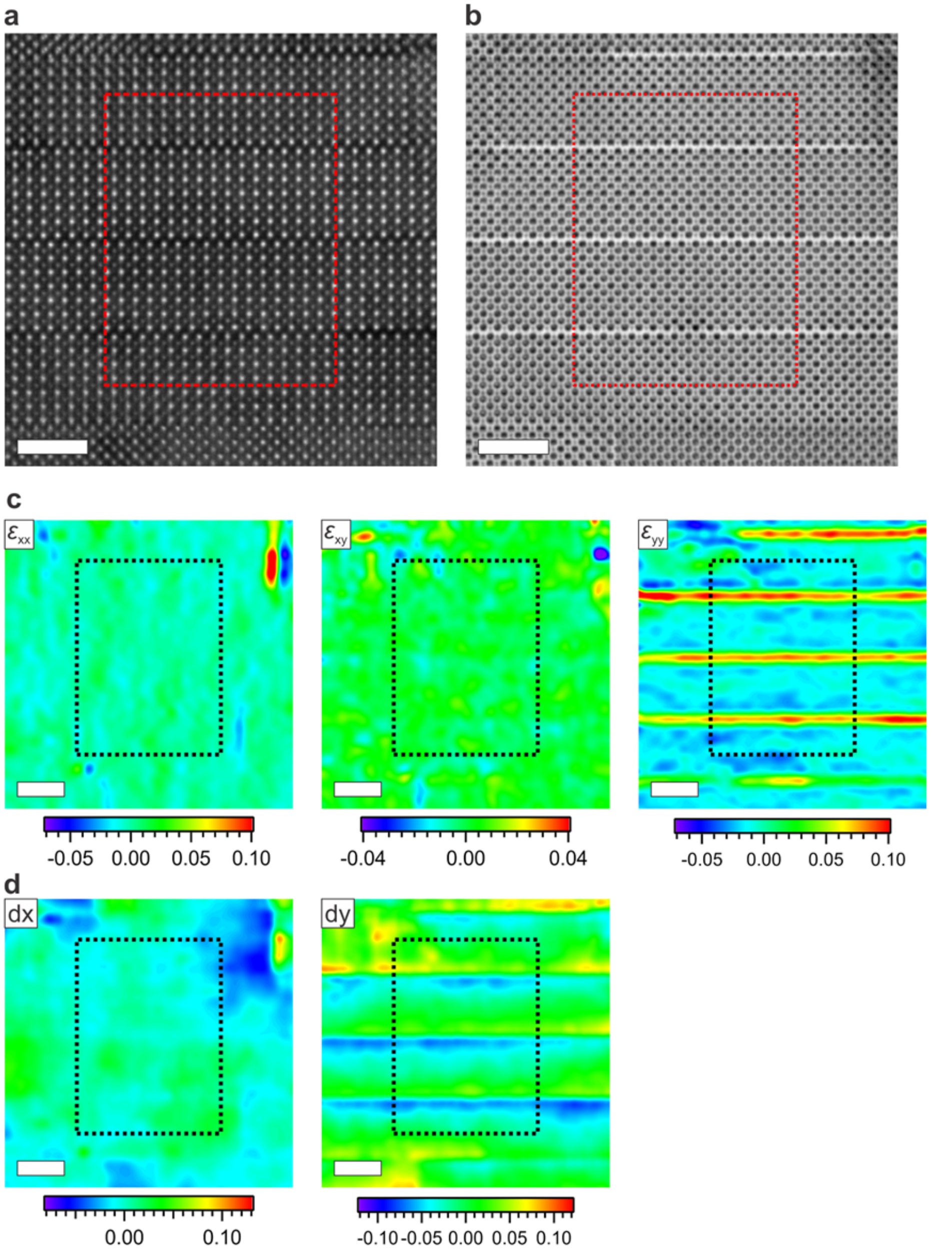

Supplementary Figure 10 | HAADF, BF, and strain maps of region #3 in the $Sr_7Ti_6O_{19}$ film. Drift corrected (**a**) HAADF and (**b**) BF images with the area of analysis highlighted by the dashed red box. (**c**) The ε_{xx}, ε_{xy}, and ε_{yy} strain maps with respect to the pseudo-cubic perovskite structure of the analysis area. The strain maps show that there are no long range structural ordering beyond the expected Ruddlesden-Popper structure in the area of analysis. (**d**) Color scale maps representing the deviation of the atomic columns (dx and dy) from an overlaid cubic grid calculated using all of the atomic columns within the HAADF image. Scale bar is 2 nm.

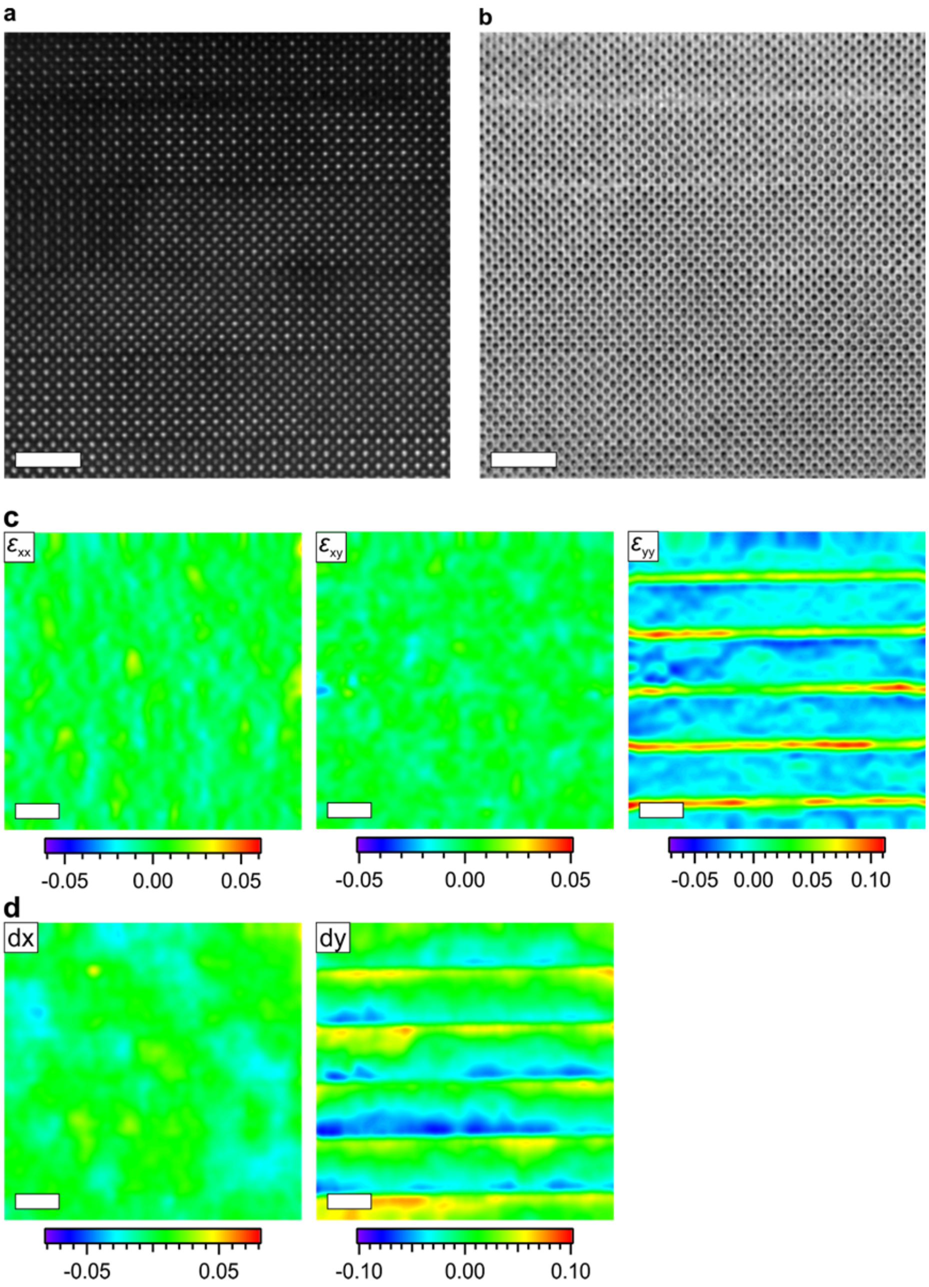

Supplementary Figure 11 | HAADF, BF, and strain maps from region #4 of the $Sr_7Ti_6O_{19}$ film. Drift corrected (**a**) HAADF and (**b**) BF images with the area of analysis highlighted by the dashed red box. (**c**) The ε_{xx}, ε_{xy}, and ε_{yy} strain maps with respect to the pseudo-cubic perovskite structure of the analysis area. The strain maps show that there are no long range structural ordering beyond the expected Ruddlesden-Popper structure in the area of analysis. (**d**) Color scale maps representing the deviation of the atomic columns (dx and dy) from an overlaid cubic grid calculated using all of the atomic columns within the HAADF image. Scale bar is 2 nm.

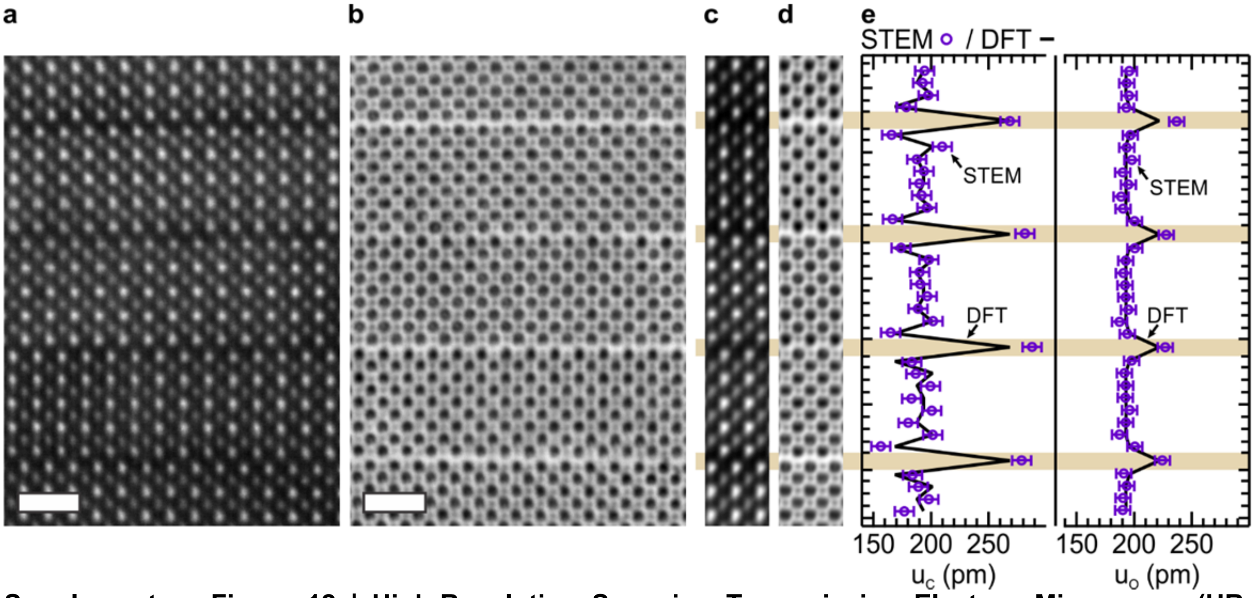

Supplementary Figure 12 | High-Resolution Scanning Transmission Electron Microscopy (HR-STEM) derived interatomic spacing of n=4 $Sr_{n+1}Ti_nO_{3n+1}$. Drift corrected (**a**) HAADF and (**b**) BF images of $Sr_5Ti_4O_{13}$ thin films on $GdScO_3$ $(101)_{Pnma}$ showing the cation and oxygen atomic columns. Scale bar is 1 nm. (**c-d**) constructed average slice of the HAADF and BF images. (**e**) HAADF and BF derived interatomic spacing of $Sr_5Ti_4O_{13}$ (open purple circles) overlapped with the DFT values (solid gray line) showing exceptional agreement between theory and experiments. The error bars are taken to be the root mean standard deviation from the positions of a best fit lattice determined by cross validation.

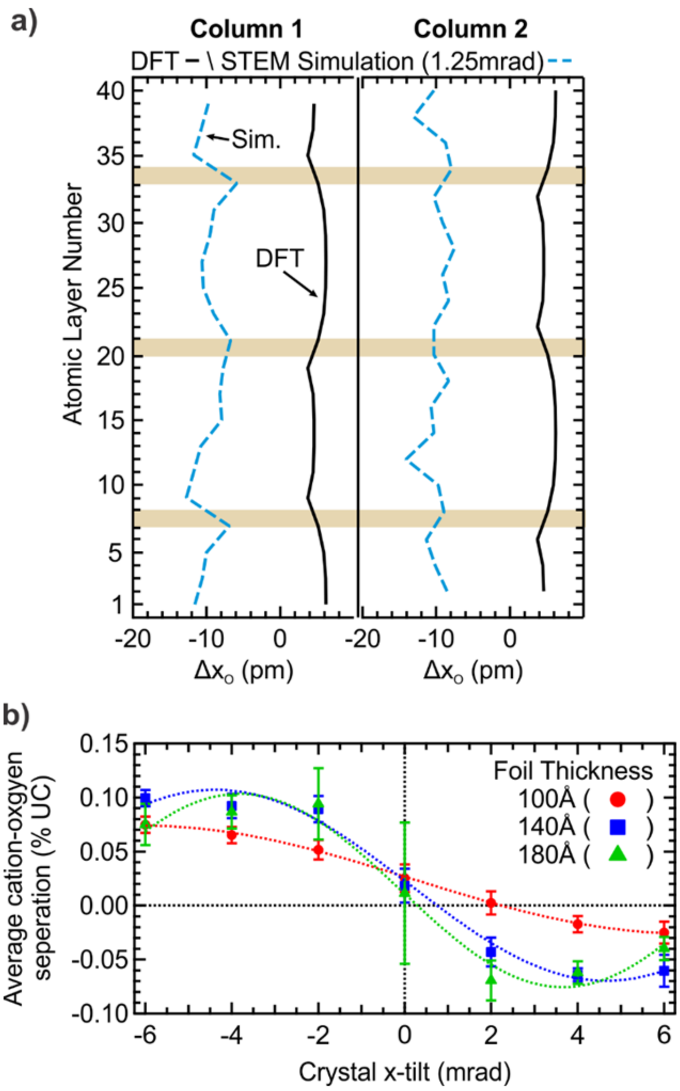

Supplementary Figure 13 | Simulated average cation-oxygen in-plane displacements. a) DFT calculated positions and simulated BF oxygen positions of the ferroelectric $Sr_7Ti_6O_{19}$ structure on $GdScO_3$ $(101)_{Pnma}$ with a sample tilt of 1.25 mrad. The simulated images show the oxygen atomic columns experience a large in-plane shift due to sample tilt. **b**) The average cation-oxygen in-plane displacements were calculated from simulated HAADF and BF STEM images for a defocus of 100 Å and different foil thicknesses, 10 nm (red circle), 14 nm (blue square) and 18 nm (green triangle). The displacements were fit with a sine function (dashed line) to visualize the trend. Cation and oxygen positions were determined from the simulated HAADF and BF images, respectively. The error bars represent the root mean squared deviation of multiple simulations at each parameter.

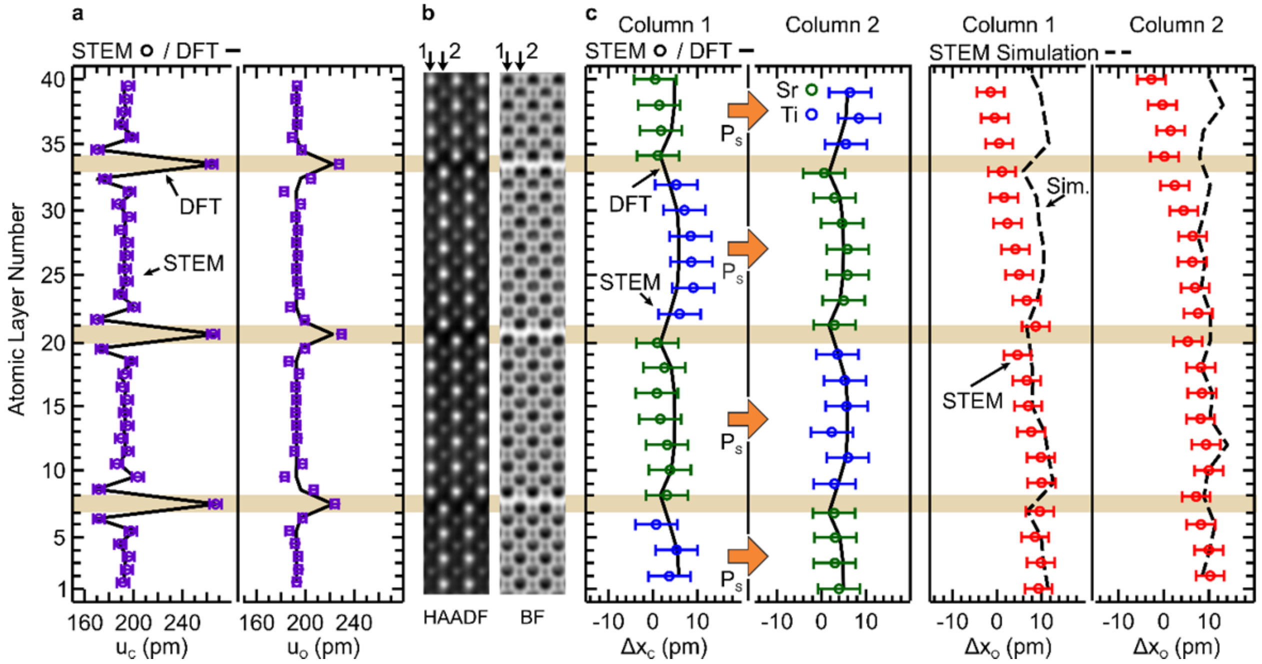

Supplementary Figure 14 | Atomic columns in $Sr_7Ti_6O_{19}$ film region #3. Cation u_c and oxygen u_o interatomic spacing (purple open circles) extracted from constructed drift corrected high angle annular dark field (HAADF) and bright field (BF) STEM images superimposed on the DFT calculated values (gray lines). The characteristic decrease in interplanar spacing in u_c and absence in u_o adjacent to the rock salt layers indicates the presence of rumpling at the rock salt layer; exceptional agreement between experiments and DFT is seen. (**b**) Constructed average HAADF and BF STEM slices, which are repeated for clarity. (**c**) Cation Δx_c (blue and green open circles) and oxygen Δx_o (red open circles) displacements along the $[100]_{PC}$ direction measured from the drift corrected HAADF and BF images. The FE DFT calculated value (solid gray lines) and the FE STEM simulated Δx_o values (dashed black line) are superimposed to show agreement between simulation and experiments. The simulated Δx_o values were done with a 1.25 mrad sample tilt around the $[001]_{PC}$ axis. The error bars are taken to be the root mean standard deviation from the positions of a best fit lattice determined by cross validation.

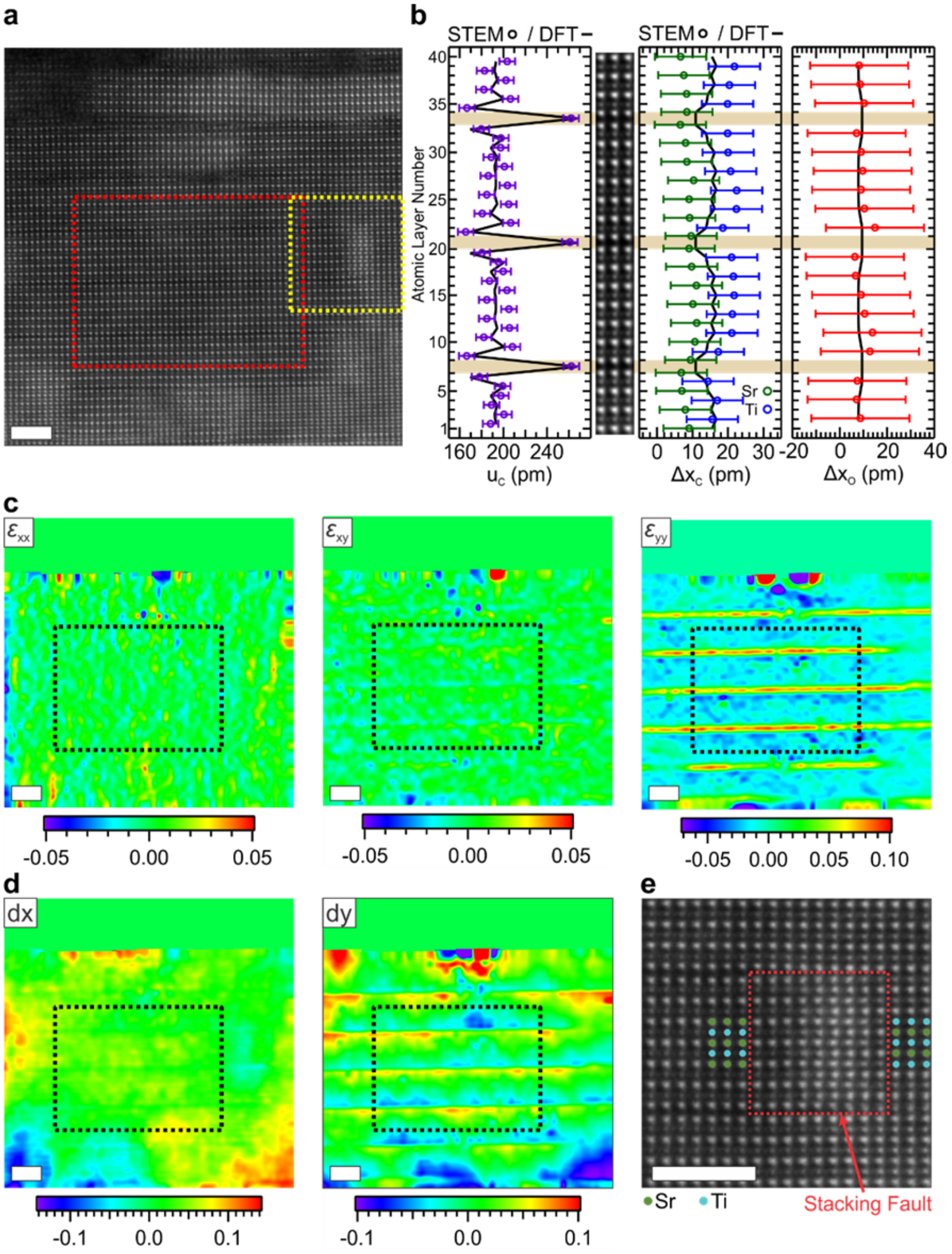

Supplementary Figure 15 | Atomic structure of $Sr_7Ti_6O_{19}$ along the <110>$_{PC}$ direction. (**a**) Drift corrected HAADF image with the area of analysis highlighted by the dashed red box and a stacking fault highlighted by the yellow box. (**b**) Cation u_c interatomic spacing (purple open circles), Δx_c (blue and green open circles), and Δx_o (red open circle) displacements along the $[100]_{PC}$ from the drift corrected ADF image superimposed on the FE DFT calculated values (gray lines). The error bars are taken to be the root mean standard deviation from the positions of a best fit lattice determined by cross validation. (**c**) The ε_{xx}, ε_{xy}, and ε_{yy} strain maps with respect to a <110>$_{PC}$ projected perovskite structure of the analysis area.

The strain maps show that there are no long range structural ordering beyond the expected Ruddlesden-Popper structure in the area of analysis (**d**) Color scale maps representing the deviation of the atomic columns (dx and dy) from an overlaid cubic grid calculated using all of the atomic columns within the HAADF image. (**e**) HAADF image zoomed in on the stacking fault region highlighted in (**a**). Because, the stacking fault plane is 45 degrees with respect to the surface, the area in the red box contains projection of two superimposed perovskite slabs, *n*=6 and *n*=19. Hence, the sharp structural features with the stacking fault as seen in Figure 5, are spread out over an area of several pseudocubic perovskite blocks. Scale bar is 2 nm. Consistent with the B-site nature of the ferroelectricity in this compound, the polar displacements of Ti cations are consistently larger than that of the Sr cations in neighboring layers. This causes the wiggle pattern in the Δx_C plot. While the qualitative agreement between DFT and experiments is certainly present, the magnitude of the experimental displacements (at room temperature) are larger than the DFT (0K calculation). The origin of this discrepancy is currently not understood.

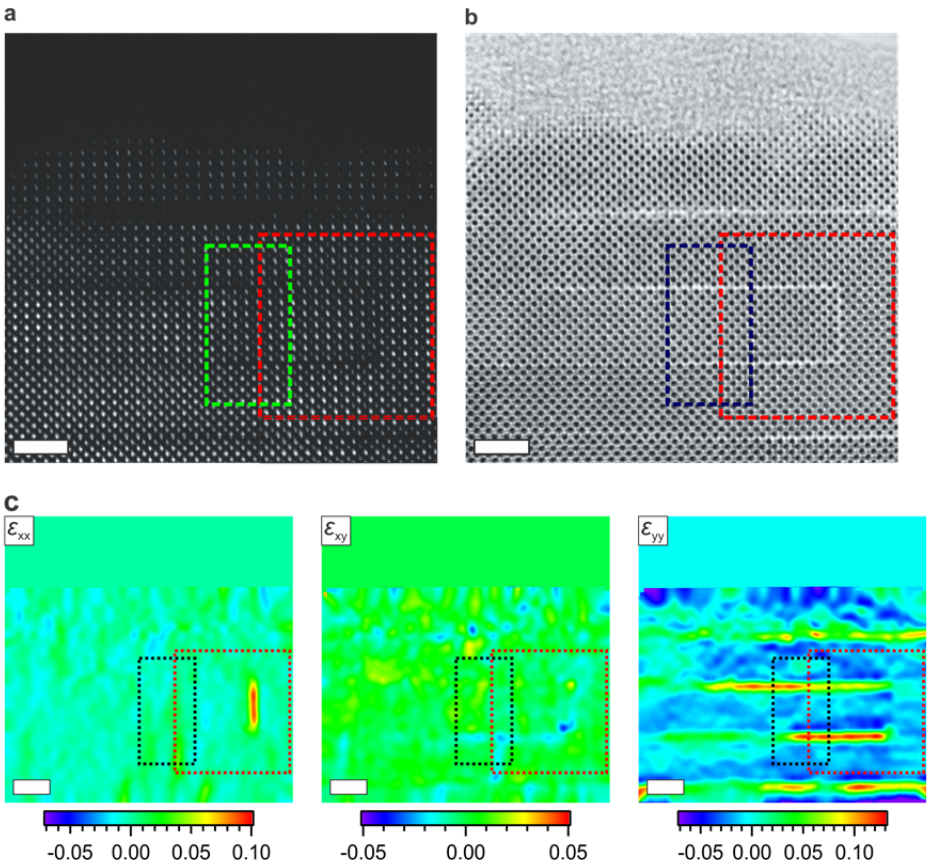

Supplementary Figure 16 | HAADF, BF, and strain maps from a region near a stacking fault Drift corrected (**a**) High angle annular dark field and (**b**) bright field images of a stacking fault in $Sr_7Ti_6O_{19}$ film on $GdScO_3$ $(101)_{Pnma}$. The reference sample area (green box) and analyzed stacking fault area (red box) are highlighted in the HAADF and BF images. (**c**) The ε_{xx}, ε_{xy}, and ε_{yy} strain maps with respect to the pseudo-cubic perovskite structure of the analysis area. Strain maps of the Scale bar is 2 nm.

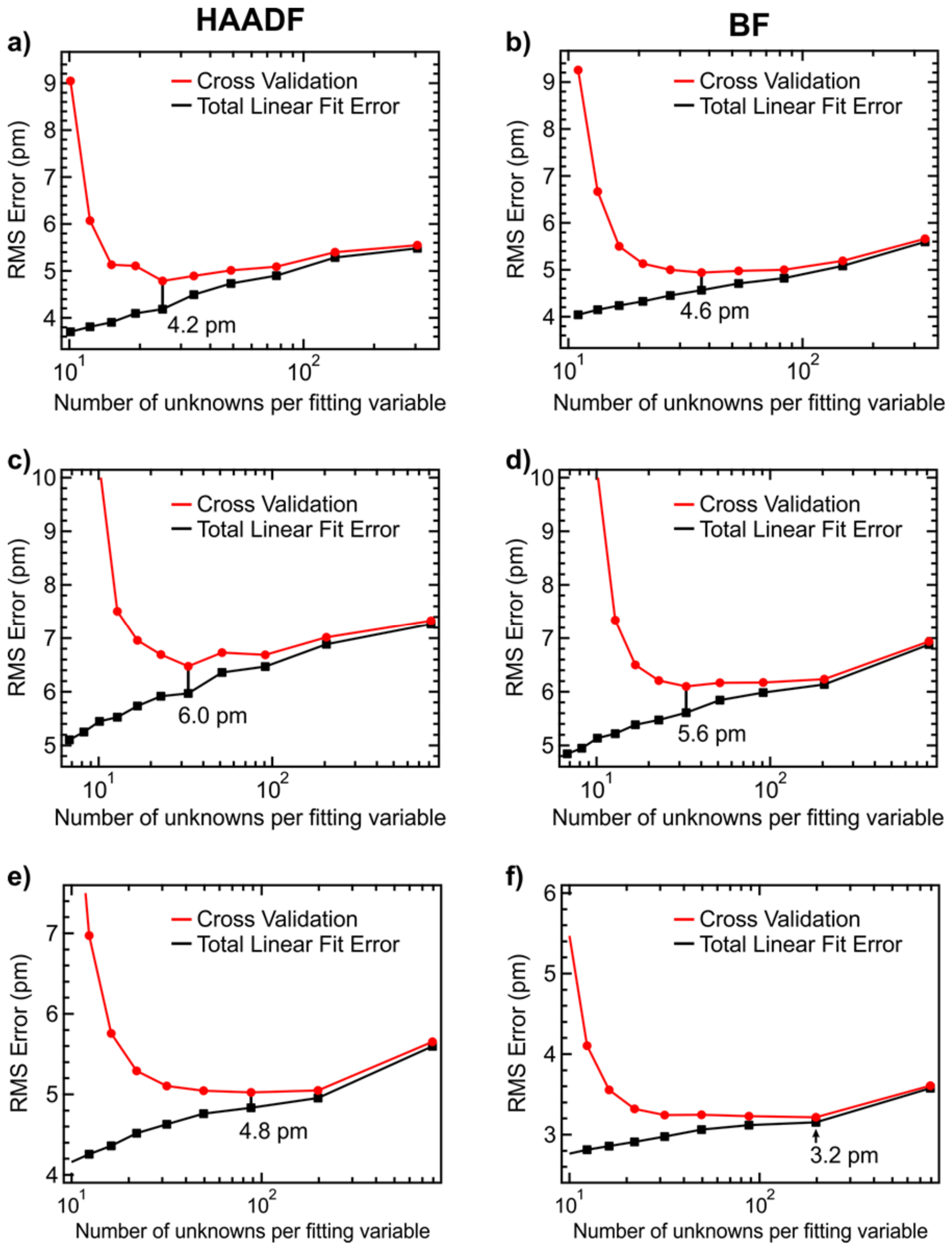

Supplementary Figure 17 | Error estimation of atomic displacement measurements. Average cross validation (CV) scores calculated from the HAADF and BF images for regions #1, #2, and #3. Using the average MRS deviation fitting error, upper bounds for the precision in our measurements are estimated to be (**a**) 4.2 pm, (**b**) 4.6 pm, (**c**) 6.0 pm, (**d**) 5.6 pm, (**e**) 4.8 pm, and (**f**) 3.2 pm for region #1 HAADF, region #1 BF, region #2 HAADF, region #2 BF, region #3 HAADF, and region #3 BF, respectively.

Supplementary Note 1 | Irreducible Representation Analysis of Relaxed Structures

We have performed an irreducible representation (irrep) analysis of the three metastable structures in the primitive unit cell as obtained from ionic relaxations in DFT. These structures correspond to the distortion patterns E_{u1}, E_{g1}, and E_{u2}, shown in Supp. Fig. 1. The dominant irreps of the distortions for these structures are Γ_{5-}, Γ_{5+}, and again Γ_{5-} respectively, with amplitudes 0.411, 0.384, and 0.327. In other words, in all of the three modes, the total ionic displacement is of similar magnitude (with a ~30% variation), but the difference in the displacement patterns leads to different energies of the metastable structures. The only other phonon irrep that these modes couple to is the fully symmetric Γ_{1+} mode. The amplitude of the Γ_{1+} displacements for the E_{1u} structure is 0.154, which is significantly larger than the A_{1g} displacements for E_{g1} (0.011) and E_{u1} (0.014) structures, which is possibly partially responsible of the larger energy gain of the E_{u1} structure.

The symmetry of these structures permit a component of strain that transform as Γ_{1+}, which does not change the symmetry, as well as a component that transforms as Γ_{4+}. However, even though this Γ_{4+} component is allowed by symmetry, it is fixed to zero by the in-plane fixed strain boundary condition we impose.

Supplementary Note 2 | Octahedral Rotations in $Sr_7Ti_6O_{19}$

Oxygen octahedral rotations are important for the polar properties of not only various perovskites but also many layered Ruddlesden-Popper compounds, such as $Ca_3Ti_2O_7$. While they are relatively weak in bulk $SrTiO_3$, and as a result are not expected to exist at room temperature in $Sr_{n+1}Ti_nO_{3n+1}$ RP's, we performed first principles calculations to predict their strength in these compounds.

We performed frozen phonon calculations for $Sr_7Ti_6O_{19}$ with a tensile strain of 1.74% and identified the structural instabilities relevant to octahedral rotations. The Brillouin Zone of the high symmetry (paraelectric) space group I4/mmm (#139) is given in **Supplementary Figure 3**. The octahedral rotations in this structure correspond to lattice distortions with k-vector (π/a, π/a, 0) in the conventional basis, which is the X point. The frozen phonon calculations are performed in a 2-formula unit (64 atom) supercell corresponding to this k-vector.

The strongest instability at the X-point is a two-fold degenerate oxygen tilt around in-plane directions, shown schematically in **Supplementary Figure 4**. This mode consists of 99% oxygen displacements, which involve mostly rotations but also a small amount of octahedron distortion, and 1% of in-plane Sr displacements. While the imaginary frequency of this mode is 70 cm^{-1}, which can be considered significant, this is misleading since this mode involves only oxygen displacements, which is by far the lightest atom in this crystal. A better measure of the strength of a static structural distortion is the force constants matrix (FCM) eigenvalue, which is the second derivative of the energy with respect to a particular lattice distortion. The FCM eigenvalue corresponding to this tilt mode is -0.30 eV $Å^{-2}$, which is an order of magnitude smaller than the FCM eigenvalue of the leading polar mode, -2.60 eV $Å^{-2}$. Thus we expect that the octahedral rotations do not have a significant effect on the polar transition.

However, while this analysis shows that octahedral rotations are too weak to significantly affect the polar properties, it does not answer the question whether they can be present at the polar phase. In order to see whether the onset of polarization suppresses any rotational instabilities, we performed frozen phonon calculations for the X point in the ferroelectric phase as well. Since polarization breaks the 4-fold

rotational symmetry (so that the x and y directions are no longer equal) the rotational instability is no longer two fold degenerate. And interestingly, the presence of the polarization does not suppress the rotational instability along one of the in-plane directions, and there is still an instability with a FCM eigenvalue of -.30 eV $Å^{-2}$.

This shows that *at zero temperature* there are octahedral rotations in $Sr_7Ti_6O_{19}$, in addition to the (anti-)polar distortions. While a finite temperature prediction of the crystal structure is beyond the scope of this study, since the FCM eigenvalue of the octahedral rotational instability is almost an order of magnitude smaller than that of the FE mode, we expect that the onset of octahedral rotations occur at a much smaller temperature. As a result, it is very unlikely to see any signature of octahedral rotations in our room temperature measurements.

Supplementary Reference

1. Lee, J. & Arias, T. A. Structural phase transitions in Ruddlesden-Popper phases of strontium titanate: Ab initio and modulated Ginzburg-Landau approaches. *Phys. Rev. B - Condens. Matter Mater. Phys.* **82,** 180104(R) (2010).
